# Growth, Electronic and Electrical Characterization of Ge-Rich Ge–Sb–Te Alloy

**DOI:** 10.3390/nano12081340

**Published:** 2022-04-13

**Authors:** Adriano Díaz Fattorini, Caroline Chèze, Iñaki López García, Christian Petrucci, Marco Bertelli, Flavia Righi Riva, Simone Prili, Stefania M. S. Privitera, Marzia Buscema, Antonella Sciuto, Salvatore Di Franco, Giuseppe D’Arrigo, Massimo Longo, Sara De Simone, Valentina Mussi, Ernesto Placidi, Marie-Claire Cyrille, Nguyet-Phuong Tran, Raffaella Calarco, Fabrizio Arciprete

**Affiliations:** 1 Istituto per la Microelettronica e Microsistemi (IMM), Consiglio Nazionale delle Ricerche (CNR), Via del Fosso del Cavaliere 100, 00133 Rome, Italy; adriano.diazfattorini@artov.imm.cnr.it (A.D.F.); chridapa@gmail.com (C.P.); marco.bertelli@artov.imm.cnr.it (M.B.); massimo.longo@artov.imm.cnr.it (M.L.); sara.desimone@artov.inm.cnr.it (S.D.S.); valentina.mussi@artov.imm.cnr.it (V.M.); 2 Dipartimento di Fisica, Università di Roma “Tor Vergata”, Via della Ricerca Scientifica 1, 00133 Rome, Italy; cheze_caroline@yahoo.fr (C.C.); friva@roma2.infn.it (F.R.R.); simone.prili@roma2.infn.it (S.P.); ernesto.placidi@uniroma1.it (E.P.); fabrizio.arciprete@roma2.infn.it (F.A.); 3 Istituto per la Microelettronica e Microsistemi (IMM), Consiglio Nazionale delle Ricerche (CNR), Zona Industriale Ottava Strada 5, 95121 Catania, Italy; inaki.lopez@outlook.com (I.L.G.); stefania.privitera@imm.cnr.it (S.M.S.P.); marzia.buscema@st.com (M.B.); antonella.sciuto@imm.cnr.it (A.S.); salvatore.difranco@imm.cnr.it (S.D.F.); giuseppe.darrigo@imm.cnr.it (G.D.); 4 Department of Physics, Sapienza University of Rome, P.le Aldo Moro 5, 00185 Rome, Italy; 5 Leti, CEA, University Grenoble Alpes, 38000 Grenoble, France; marie-claire.cyrille@cea.fr (M.-C.C.); nguyet-phuong.tran@cea.fr (N.-P.T.)

**Keywords:** PCM, Ge-rich GST alloys, Raman, electronic properties

## Abstract

In this study, we deposit a Ge-rich Ge–Sb–Te alloy by physical vapor deposition (PVD) in the amorphous phase on silicon substrates. We study in-situ, by X-ray and ultraviolet photoemission spectroscopies (XPS and UPS), the electronic properties and carefully ascertain the alloy composition to be GST 29 20 28. Subsequently, Raman spectroscopy is employed to corroborate the results from the photoemission study. X-ray diffraction is used upon annealing to study the crystallization of such an alloy and identify the effects of phase separation and segregation of crystalline Ge with the formation of grains along the [111] direction, as expected for such Ge-rich Ge–Sb–Te alloys. In addition, we report on the electrical characterization of single memory cells containing the Ge-rich Ge–Sb–Te alloy, including I-V characteristic curves, programming curves, and SET and RESET operation performance, as well as upon annealing temperature. A fair alignment of the electrical parameters with the current state-of-the-art of conventional (GeTe)_n_-(Sb_2_Te_3_)_m_ alloys, deposited by PVD, is found, but with enhanced thermal stability, which allows for data retention up to 230 °C.

## 1. Introduction

In recent years, the interest of the industrial and research sectors has been increasingly directed towards the so-called “Internet of Things” (IoT) [1]. The IoT is a new way of conceiving the world around us, and it aims to connect people with each other, people with things, and things together. One of the fields in which IoT is most widespread is certainly the automotive one, the purpose of which is the development of car automation, in order to improve safety, emissions, and production costs. The idea is to be able to connect cars with each other, as well as with road devices, buildings, cyclists, and even pedestrians, all this to make driving safer. To reach this level of intelligent driving, it is necessary to have devices capable of acquiring, storing, and sending data, while being cheap, fast in reading and writing, non-volatile, and resistant over the years. This is one of the biggest challenges in this sector. Phase change materials (PCM) are the materials of choice for such applications [2]. The peculiarities of PCM-based memory devices are a low-current for the phase transition, good reading and writing speed, and low production costs. Among the materials most used in such devices, there is (GeTe)_n_-(Sb_2_Te_3_)_m_ (GST), a ternary alloy consisting of two binary compounds, GeTe and Sb_2_Te_3_, especially in the composition Ge_2_Sb_2_Te_5_ (GST225) [3]. The use of GST for automotive is a great challenge, above all because, in some parts of the vehicles, temperatures up to 300 °C can be reached (in the vicinity of the exhaust gases even 800 °C) also in conditions of stationary operations. Therefore, the devices must be able to work at high temperatures (at least 160 °C) to keep the data stable for at least ten years [4]. One of the disadvantages of using GST225 is its crystallization temperature (about 150 °C), which does not favor good thermal stability and, therefore, data retention for automotive applications. When considering materials for automotive applications, it must also be borne in mind that the memories undergo, for short times, end-of-process treatments, which occur at around 400 °C. In recent years, several attempts to improve the crystallization temperature (T_x_) were made by doping the GST sample with C [5,6], O [7,8], N [9,10], or Ge itself, the so-called Ge-rich GST [11,12]. The T_x_ of Ge-rich GST was demonstrated to increase almost linearly with Ge content and has proven data retention after soldering reflow in industrial integrated device [11]. Nevertheless, as very recently pointed out by Redaelli et al. [13], although Ge-rich GST solved the issue of high temperature retention requests, a large degree of material management, in terms of material instabilities, remains. Finally, it is worth mentioning that In–Sb–Te and In–Ge–Te phase-change alloys are also of interest, since, for high indium contents, they exhibit higher thermal stability of the amorphous phase, with respect to the Ge–Sb–Te alloys. The crystallization temperature is increased up to 290 °C, in the case of In_3_Sb_1_Te_2_ [14], and 276 °C, in the case of doped In–Ge–Te, for which 10-years retention at temperatures higher than 150 °C has been found [15]. Reports on In-based alloys, both in the form of planar PCM devices [16] and as single and core-shell nanowires [17,18], have been published. Therefore, it is important to investigate the possibility of using appropriate material alloys and combinations to improve on material management issues.

In this work, we present an extensive characterization of Ge-rich GST, of composition GST 29 20 28, with the assessment of its quality. Electronic and compositional properties are investigated by photoemission characterization, vibrational by Raman spectroscopy, and structural by X-ray diffraction. The electrical characterization confirmed the high working temperature in single memory devices, obtained using a GST225 buffer layer underneath the Ge-rich GST. The novelty of the present work is the usage of such a buffer layer, which improves the electrical bottom contact and guarantees the thermal stability typical of Ge-rich GST.

## 2. Experimental

### 2.1. Sample Growth

The PCM films were deposited on Si(001)/SiO_2_ substrates via physical vapor deposition (PVD) in a custom-made, ultra-high vacuum (UHV) chamber system, equipped with Te, Sb, and Ge (Alfa Aisar, Haverhill, MA, USA) loaded in Knudsen cells (Dr. Eberl MBE-Komponenten GmbH, Weil der Stadt Germany) for thermal evaporation. The pressure during the growth reached high 10^−9^ Torr. In order to grow completely amorphous films, the substrate temperature was kept at nominal room temperature (RT). However, due to radiative heating from the cell crucibles, the substrate temperature was actually in the range 85–90 °C. Thus, some faint crystallization might be obtained. The fluxes ratios were kept to Ge:Sb:Te = 1:1:3 and Ge:Sb:Te = 2.5:1:1.8, respectively, for achieving nominal GST225 and Ge-rich GST alloy compositions. The growth time was 17 and 30 min for a nominal deposited thickness of 24 nm for both GST films. The same growth conditions were used for the realization of a GST225 (24 nm)/Ge-rich GST (24 nm) double-layer heterostructure on a single-cell vehicle for electrical testing.

### 2.2. Photoemission Characterization

Photoemission spectra were acquired using a UHV chamber, dedicated to X-ray and ultraviolet photoelectron spectroscopy (XPS and UPS) and connected in UHV to the PVD growth chamber. XPS spectroscopy was performed using an Omicron DAR 400 Al/Mg Kα non monochromatized X-ray source (Taunusstein, Germany). To collect and analyze photoelectrons, a 100 mm hemispherical VG-CLAM2 electron spectrometer (Uckfield, UK) with a single channeltron and 4 mm entrance slit was used. XPS experiments were performed with a pass energy E_pass_ = 20 eV, which means an instrumental resolution of ∆E = 0.4 eV, to be convoluted with the natural linewidth of the sources: ∆E_Al_ = 0.84 eV and ∆E_Mg_ = 0.68 eV. In order to have the best possible resolution, all the XPS spectra were collected by using the Mg Kα radiation, except the one around the Ge 2p core levels, which would be superimposed on the secondary electrons background tail, for which, we used the Al Kα radiation. UPS (Uckfield, UK) experiments were performed with a pass energy E_pass_ = 1 eV, which means an instrumental resolution of ∆E = 0.02 eV to be convoluted with the natural linewidth of the sources: ∆E_HeI_ = 0.003 eV. All XPS spectra were analyzed and fitted by means of the KolXPD software (version 1.8.0) and libraries (http://kolxpd.com accessed on 8 August 2018) using Voigt peaks and a Shirley background.

### 2.3. X-ray Diffraction

X-ray diffraction (XRD) measurements were performed ex-situ after the deposition of a 10 nm thick protective W capping layer and successive annealing for 15 min at increasing temperatures, from 30 to 400 °C. XRD was carried out using a Bruker (Billerica, MA, USA) D8 Discover diffractometer, equipped with a Cu X-ray source and Anton Paar DHS1100 (Graz, Austria) dome-type heating stage for temperature measurements in N_2_ atmosphere. Grazing incidence diffraction (GID) scans, after final cooling at RT, were acquired under an angle of 0.8°.

### 2.4. Raman

A Raman imaging DXR2xi microscope from Thermo Scientific (Waltham, MA, USA) was used to carry out the Raman measurements. The operation of the device is based on data acquisition, carried out in “backscattering” geometry, using a green laser beam with wavelength λ = 532 nm, as well as acquiring the data with a 50× magnification objective. For all the measurements carried out, the same experimental conditions were used, namely exposure time (t = 0.1 s), number of acquisitions (n = 200), and laser power on the sample (P = 4 mW).

### 2.5. Memory Device Realization

A classical mushroom architecture was selected to verify the memory functionalities. A cylindrical heater in titanium nitride was used as the back contact, and a tungsten layer, defined by lift-off procedure, was used as the top contact. The chalcogenide layer was laterally defined through plasma etching in an inductively coupled plasma system, with a gas mixture of CHF_3_:Ar (25:30 sccm) having a radio frequency power of 200 W and bias of 75 V. The hard mask was fabricated in hydrogen silsesquioxane resist (Dow Corning^®^ (Midland, MI, USA) XR-1541 e-beam resist), patterned by electron beam lithography in an E-Line Raith apparatus (Dortmund, Germany). The same e-beam equipment was used to inspect the memory devices used in the modality scanning electron microscope.

### 2.6. Electrical Measurements

The electrical characterization of PCM devices has been performed by using a Keysight (Santa Rosa, CA, USA) MSO64B oscilloscope and Keysight 81110A pulse pattern generator. The first RESET operation was achieved by applying a train of 300 ns pulses with increasing voltage (staircase up). Each programming pulse has been followed by a reading pulse at lower voltage (0.2 V). After reaching a maximum voltage, determined as the voltage at which the device switch is observed, the voltage pulse is decreases (staircase down).

## 3. Results and Discussion

**Figure 1:**
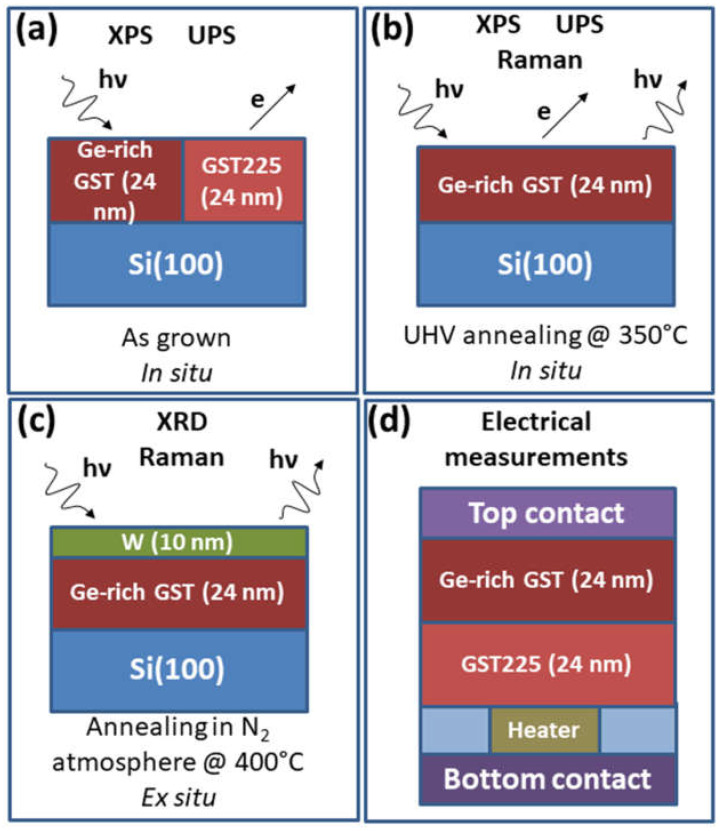
Schematics of samples and experiments carried out within this work: (**a**) XPS and UPS experiment on as grown sample; (**b**) XPS, UPS, and Raman measurements on as grown sample, after UHV annealing at 350 °C; (**c**) XRD and Raman investigation after crystallization, by means of annealing at 400 °C in N_2_ atmosphere; (**d**) electrical measurements of the GST225/Ge-rich GST memory devices.

In Figure 1, we show a schematic of the samples and experimental processes presented in the following of the present work. Photoemission spectroscopy was used in situ for the identification of elements near the surface of the Ge-rich GST, obtaining information on the local chemical environments and valence band electronic structure. The stoichiometry and concentration of elements were also obtained by a quantitative analysis of the XPS data. This spectroscopy is also a valuable tool for providing information on phase separation. In Figure 1a, the schematic for the XPS and UPS experiment is provided.

**Figure 2:**
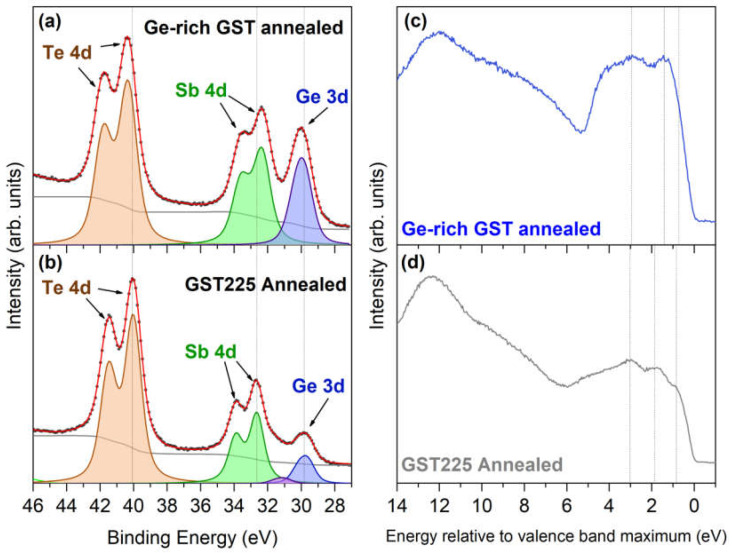
XPS spectra of (**a**) Ge-rich GST and (**b**) GST225 after annealing. Experimental data are presented using black dots, while red and gray lines correspond to the total data fit and Shirley background. Contributions from plasmon excitations, Kα_3_ and Kα_4_ lines are omitted for clarity. UPS spectra of (**c**) Ge-rich GST and (**d**) GST225 after annealing.

In Figure 2, we report the comparison between the shallow core levels of (a) Ge-rich GST and (b) GST225 sample, collected after annealing for 30 min in UHV at 350 °C (see Figure 1b). 

From the analysis of the shallow core levels, we calculated the composition of the alloys obtaining Ge_18_Sb_26_Te_56_, compatible with GST 14 20 43, as well as Ge_38_Sb_26_Te_36_, compatible with GST 29 20 28, for GST225 and Ge-rich GST, respectively. A comparison between the binding energy (BE) of the core levels, as determined by the fitting of the XPS spectra, revealed that the Sb 4d doublet of Ge-rich GST shifts to BE lower than that of GST225; conversely, Te 4d and Ge 3d core levels both move at higher BE, compared to GST225. The general trend of the chemical shifts, observed in the XPS spectra, reflects the differences in the composition between the two alloys, as already observed, in the case of Ge-rich GST-based heterostructure, as reported in this special issue [19]. A chemical shift of the core levels, after changing the GST alloy composition, is an initial state effect, which is related to a change in chemical bonding [20,21,22]. After the formation of a ternary Ge–Sb–Te alloy, the BE of Te 4d core levels should be in between the values expected for metallic Te and Sb_2_Te_3_. The more the alloy is Ge-rich, the more Te 4d core levels are shifted towards higher BEs. The same holds for Ge 3d core levels. In the case of Sb, 4d levels shift towards lower BEs are expected. The BE shifts observed in our samples agree with this general trend, if we consider the expected absolute BE positions for pure Te 4d, Sb 4d, and Ge 3d core levels (40.6 eV, 32.1 eV, and 29.3 eV, respectively).

Furthermore, from the inspection of the UPS spectra in Figure 2c,d a clear difference in the valence band (VB) line shape can be seen. We could identify the shallow core levels Te 5s at about 12 eV, as well as Sb 5s and Ge 4s, located in the region between 10 and 6 eV, respectively. Therefore, it is evident that s electrons are quite deep and do not participate in the valence band structure. The proper valence band is observed between 6 and 0 eV and is a mix of Te 5p, Sb 5p, and Ge 4p states. In particular, the GST225 VB is characterized by three main features, at 0.85, 1.87, and 3.03 eV, as well as a broad structure extending from 1 to 6 eV, compatible with the VB of crystalline GST225 [21,23,24,25] and observed for a GST225/GST-based heterostructure [19]. In addition, the Ge-rich GST spectrum shows a shoulder at 0.72 eV, a clear peak at 1.41 eV, and broad double feature in the range 3–4 eV, typical of amorphous Ge-rich GST alloys. A comparison with literature calculation is pretty well in agreement [26]. The UPS analysis suggests that the Ge-rich GST sample is predominantly amorphous, even after annealing in UHV, as already observed by the XPS data analysis. This finding is evidence that the sample is amorphous, even after annealing in UHV. It is important to note that crystallization in UHV is not straightforward and strongly depends on the GST composition. In a recent paper, we proved efficient annealing in UHV of GST225 films. Indeed, the T_x_ for Ge-rich GST is expected to be higher than for GST225, so that an annealing temperature of 350 °C was used in the attempt to crystallize Ge-rich GST; such treatment might have induced desorption, instead of crystallization.

Theoretical calculations on the stability of Ge-rich GST have been carried out by O. Abou El Kheir et al. using density functional theory [26,27], showing that amorphous Ge-rich GST alloy is metastable, while its crystalline form is expected to be extremely unstable and decomposes in different alloys. Among others, the formation of GST323 is very likely. The high T_x_ is due to the segregation of crystalline Ge upon crystallization of the amorphous phase [28]. The mass transport involved in this phase separation slows down the crystallization kinetics, which leads to a higher T_x_.

**Figure 3:**
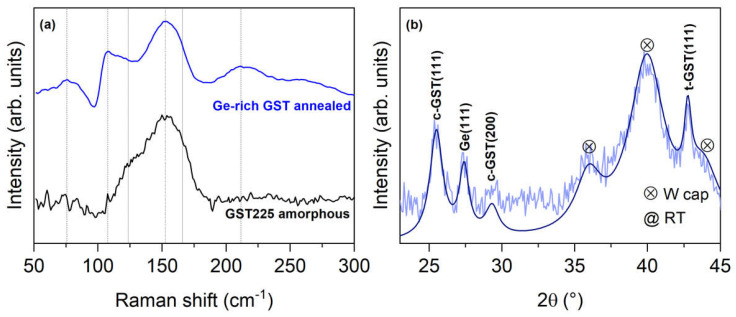
(**a**) Raman spectra of the Ge-rich GST and GST225 alloy. Dashed vertical lines are a guide to the eye for the main features; (**b**) 2θ GID curve of the W/Ge-rich GST/SiO_2_/Si(001)stack, acquired at RT, after crystallization up to 400 °C. Light blue: experimental data; dark blue: fitted curve, using Lorentzian peaks.

In Figure 3a, we present the Raman characterization of the Ge-rich GST sample, after removing it from the UHV (where it was subjected to annealing), compared with a reference amorphous GST225 alloy. Two peaks, at 106 and 154 cm^−1^, and a broad peak, at 60–80 cm^−1^, are visible. The latter peak is positioned at the same shift of the A_1g_(1) mode of Sb_2_Te_3_ [29]. However, unlike GST225, Ge-rich GST displays a sharp edge at 106 cm^−1^, followed by a decreasing slope at the same position of the E_g_ mode of crystalline GST225, thus, possibly indicating a very faint crystallization of the Ge-rich GST, due to the annealing procedure. The peak at 123.6 cm^−1^, which is present in GST225, is less prominent for the Ge-rich GST alloy [30]. Furthermore, in Ge-rich GST, a broad feature at 215 cm^−1^, typical of the stretching of the Ge–Ge bond in the tetrahedral configuration is present [31]. We also note the absence of bands assigned to the TO and LO vibrational modes of amorphous Ge, usually present from 190 to 300 cm^−1^ [32], suggesting that the UHV annealings were not sufficient to enable the segregation of Ge in the amorphous phase. Furthermore, the absence of any additional peak at around 300 cm^−1^, to be ascribed to pure crystalline Ge, is the indication that the excess of Ge was not crystallized at the temperature used for the annealing performed in the UHV system. Therefore, even from Raman investigations, we do not have an indication of a full crystallization of both Ge-rich GST and Ge [33,34]. Our interpretation is compatible with a recent discussion on the Ge-rich GST crystallization by L. Prazakova et al. [35]. They studied, by Raman spectroscopy and XRD techniques, the crystallization mechanism of Ge-rich GST alloys with a large Ge enrichment (15–55 at. %), where the structural evolution process in temperature begins with the Ge–Te bond rearrangement around stable SbTe structural units, independently of the Ge content in the alloy.

From XPS measurements, we calculated the actual composition of the deposited Ge-rich GST to be GST 29 20 28, as obtained from flux ratios Ge:Sb:Te = 2.5:1:1.8. The percentage of the deposited Ge is 37.6%; therefore, it is important to investigate the segregation of Ge, as well as of the Ge-rich GST decomposition, as recently reported by Cecchi et al. [36]. Furthermore, we learned from Raman measurements that the excess of amorphous Ge is not segregating in the as-grown samples, thus ensuring that the deposition of amorphous GST 29 20 28 layers in devices will be homogeneous.

To crystallize the sample and observe the expected phase separation [13], we resorted to annealing in the N_2_ atmosphere from RT to 400 °C, at steps of 50 °C, waiting 15 min at each step (see Figure 1c). The 2θ° GID curve shown in Figure 3b is acquired after cooling down to room temperature. Several diffraction peaks can be observed. The three peaks corresponding to the W capping layer are marked by crossed circles. The peaks at θ = 25.52° and 29.36° are identified as (111) and (200) Bragg reflections of the GST in the cubic crystalline phase, while the peak θ = 42.77° is ascribed to the trigonal crystalline phase. Interestingly, the peak at θ = 27.44 is the contribution of the (111) reflection of Ge, showing the Ge segregation upon annealing [37]. An additional consideration is on the amount of crystallized Ge that results rather small, as inferred from the overall intensity of the XRD and, more evidently, Raman peaks. Again, in this case, our observations are compatible with the results from L. Prazakova et al. [35], who reported that the crystallization of the cubic GST phase is followed by the heterogeneous nucleation and consequent growth of the Ge crystalline phase. This sequential crystallizations have been well-observed in layers with low Ge content, since they take place at different temperatures. On the contrary, the two processes appear simultaneously at high temperatures in the layers with high Ge content, resulting from delayed GST phase crystallization and fast Ge crystal growth.

Our annealing and XRD data provided the information (data not shown here) that the crystallization occurred completely at 350 °C; hence, it is important to know the annealing temperature of memory devices prior to the forming step (see below). The observation that both trigonal and cubic lattices coexist in the crystalline phase after annealing, thus demonstrating that the annealing of Ge-rich GST is not sufficient to transform the whole volume into the trigonal phase, is rather interesting. Such a finding will be the subject of further investigation on several Ge-rich GST samples with different compositions.

**Figure 4:**
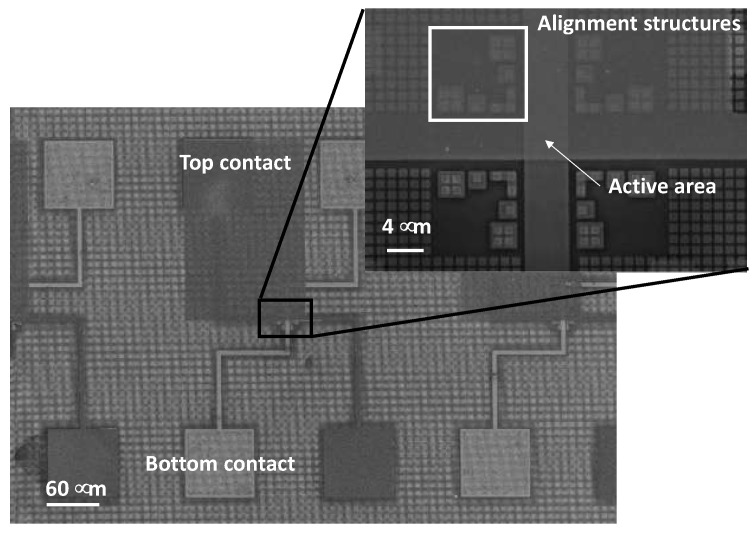
SEM microscope image of the memory devices. Bottom and top contacts are clearly visible. The blow-up shows a detail with evident alignment markers of the area of crossing of the bottom and top contacts, where the active material deposited (GST225/Ge-rich GST) is present, together with the nanometric TiN heater structure.

To further evaluate the material quality of the Ge-rich GST material for electrical memory applications, a duplicate sample was prepared in a device structure. Figure 4 shows a secondary electron microscopy (SEM) image of the top contact of the fabricated devices.

The Ge-rich GST sample was fabricated on a GST225 amorphous buffer of 24 nm (see Figure 1d). Such a buffer was deposited to obtain a better contact with the bottom of the device; as for Ge-rich compositions, we expect to have a higher resistivity. We also performed measurements of devices with GST225 as the active material, for which the succesful annealing temperature in the nitrogen atmosphere was 180 °C for 30 min. For GST225/Ge-rich GST, the annealing temperature of 180 °C was not sufficient to produce crystallization. Therefore, according to our structural characterization, the devices were annealed at 350 °C, in order to obtain the full crystallization of the phase change layer. Figure 3 shows the main results of GST225/Ge-rich GST devices with a heater diameter of 100 nm.

**Figure 5:**
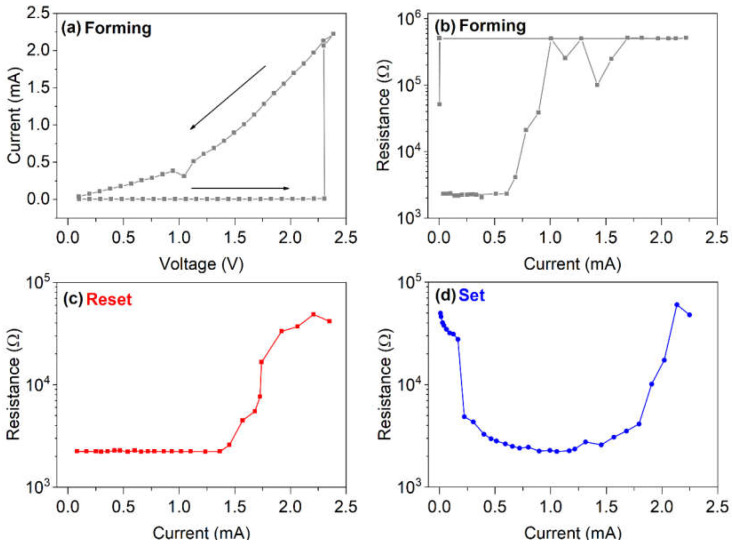
Current–voltage characteristics (**a**), resistance measured after each pulse versus programming current (**b**), as acquired during forming of the GST225/Ge-rich GST devices. Programming window of RESET (**c**) and SET (**d**) processes.

Figure 5a,b shows the current–voltage characteristics obtained during the staircase, up and down, and resistance measured, as a function of programming current, respectively. The I-V curve displays a threshold voltage V_TH_ = 2.3 V. The resistance is initially high and, after the “forming” process, it decreases during the staircase down. The forming process is suggested for Ge-rich GST materials. After forming, the device is programmed from SET to RESET (as shown in Figure 5c), from RESET to SET, and, again, to RESET, in order to determine the programming window, as shown in Figure 5d.

**Figure 6:**
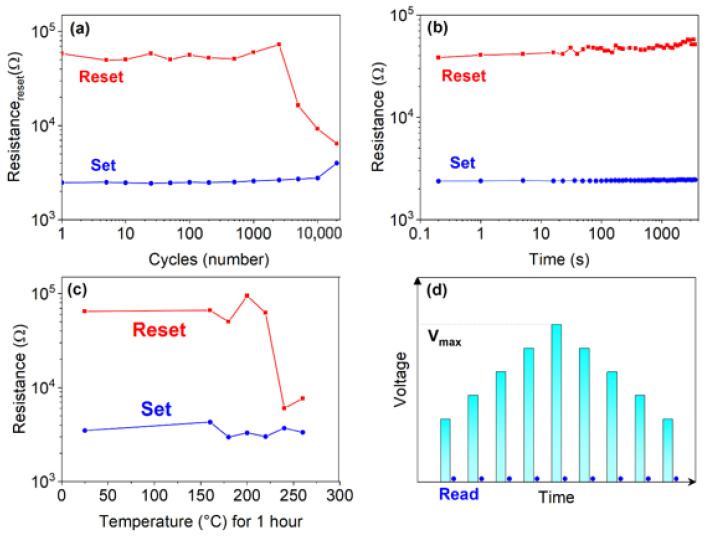
Resistance of the GST225/Ge-rich GST devices: upon cycling (**a**); versus time (**b**); after 1 h annealing at different temperatures (**c**); scheme of measurements staircase, up and down (**d**).

Figure 6 shows, as main reliability figures of merit, the SET and RESET resistance measured upon cycling (a) and resistance drift, as a function of time, in (b). The programming window resulted in more than one order of magnitude. The best measured endurance value was 2 × 10^4^ cycles. The resistance of the devices in the RESET or SET state after 1h annealing, as a function of temperature, is reported in Figure 6c. The devices are able to retain information up to 230 °C. The presence of GST225 does not adversely affect the thermal stability. Typically, the GST225 device is not able to retain the information up to 230 °C for 1 h. Upon forming, it is reasonable to assume that some interdiffusion between the two layers, with migration of Ge atoms toward the GST225, might occur with the formation of an alloy with intermediate properties. In fact, the crystallization temperature is about 350 °C, while retention is only 230 °C. Figure 6d reports the scheme of staircase, up and down, used to measure the devices and position at which the read operation was performed.

Phase change memory devices manufactured with a single Ge-rich GST layer have been studied in literature [11,13,38,39]. Despite the high tendency of Ge to segregate, alloys with Ge contents of 20–45% or more in excess, compared to the GST225 stoichiometry, are preferentially adopted, since the high Ge content may guarantee higher crystallization temperatures [11] However, the improved thermal stability is usually associated with some undesirable effects. One of these is the increase of the resistivity of the crystalline phase, increasing as the Ge content increases, as well as the corresponding increase of the drift coefficient, even in the SET state [39]. According to Ref. [39], single-cell memories with GST with an excess Ge of 35% (namely a composition close to the one adopted in this paper) exhibit a drift coefficient ν of 0.02 in the low resistance state, higher than that of GST225 (ν = 0.005). The resistance drift of both the SET and RESET states in the devices here presented is comparable to that typically measured in GST225. It is important to note that, although the thickness of the GST225 is comparable to that of the Ge-rich GST, and, even assuming some intermixing, the larger overall amount of Ge is dominating the device behavior, in terms of thermal stability. Therefore, data indicate that the presence of a GST225 buffer layer can successfully mitigate the increased resistance drift, while assuring data retention up to 230 °C (Figure 6c).

## 4. Conclusions

In this work, we present a full characterization of a PVD grown amorphous Ge-rich GST alloy. We evaluated the composition of the alloy to be Ge_36_Sb_26_Te_38_, compatible with GST 29 20 28. Although annealing in UHV was conducted up to 350 °C, no crystallization of the film occurred. UPS measurements revealed a good agreement between the experimental spectra and theoretical calculations from the literature [26].

A segregation of crystalline Ge was observed, as well as the coexistence of the trigonal and cubic lattices in the crystalline phase, after annealing of Ge-rich GST.

The investigated Ge-rich GST alloy was integrated into a PCM single-cell testing vehicle, allowing for electrical characterizations; a V_TH_ = 2.3 V and programming window of about two orders of magnitude can be obtained. The resistance drift and endurance (2 × 10^4^ cycles) turned out to be comparable to that of GST225. The electrical characterizations showed that the Ge-rich GST alloy works in a similar fashion as conventional GST225-based devices, except for the fact that the former retain the information up to 230 °C. The data retention of 230 °C, after 1 h annealing, is markedly higher than that of GST225 (about 160 °C), but lower than expected, according to the crystallization temperature (350 °C). We suggest that interdiffusion occurs during the first melting (the forming process), with migration of Ge atoms toward the GST225 and the formation of an alloy with intermediate properties.

## References

[1] Bandyopadhyay D., Sen J. (2011). Internet of Things: Applications and Challenges in Technology and Standardization. Wirel. Pers Commun..

[2] Cappelletti P., Annunziata R., Arnaud F., Disegni F., Maurelli A., Zuliani P. (2020). Phase Change Memory for Automotive Grade Embedded NVM Applications. J. Phys. D Appl. Phys..

[3] Wuttig M., Yamada N. (2007). Phase-Change Materials for Rewriteable Data Storage. Natur. Mater..

[4] Fantini A., Perniola L., Armand M., Nodin J.F., Sousa V., Persico A., Cluzel J., Jahan C., Maitrejean S., Lhostis S. Comparative Assessment of GST and GeTe Materials for Application to Embedded Phase-Change Memory Devices. Proceedings of the 2009 IEEE International Memory Workshop.

[5] Hubert Q., Jahan C., Toffoli A., Navarro G., Chandrashekar S., Noe P., Blachier D., Sousa V., Perniola L., Nodin J.-F. Lowering the Reset Current and Power Consumption of Phase-Change Memories with Carbon-Doped Ge_2_Sb_2_Te_5_. Proceedings of the 2012 4th IEEE International Memory Workshop.

[6] Li T., Shen J., Wu L., Song Z., Lv S., Cai D., Zhang S., Guo T., Song S., Zhu M. (2019). Atomic-Scale Observation of Carbon Distribution in High-Performance Carbon-Doped Ge _2_ Sb _2_ Te _5_ and Its Influence on Crystallization Behavior. J. Phys. Chem. C..

[7] Kikuchi S., Oh D.Y., Kimura I., Nishioka Y., Ueda M., Endo M., Kokaze Y., Suu K. Preparation of Oxygen-Doped and Nitrogen-Doped Ge-Sb-Te System Thin Film for Phase Change Random Access Memory by RF Magnetron Sputtering. Proceedings of the 2006 7th Annual Non-Volatile Memory Technology Symposium.

[8] Privitera S., Rimini E., Zonca R. (2004). Amorphous-to-Crystal Transition of Nitrogen- and Oxygen-Doped Ge_2_Sb_2_Te_5_ Films Studied by in Situ Resistance Measurements. Appl. Phys. Lett..

[9] Lai Y., Qiao B., Feng J., Ling Y., Lai L., Lin Y., Tang T., Cai B., Chen B. (2005). Nitrogen-Doped Ge_2_Sb_2_Te_5_ Films for Nonvolatile Memory. J. Elec. Mater..

[10] Horii H., Yi J.H., Park J.H., Ha Y.H., Baek I.G., Park S.O., Hwang Y.N., Lee S.H., Kim Y.T., Lee K.H. A Novel Cell Technology Using N-Doped GeSbTe Films for Phase Change RAM. Proceedings of the 2003 Symposium on VLSI Technology. Digest of Technical Papers (IEEE Cat. No.03CH37407).

[11] Zuliani P., Varesi E., Palumbo E., Borghi M., Tortorelli I., Erbetta D., Libera G.D., Pessina N., Gandolfo A., Prelini C. (2013). Overcoming Temperature Limitations in Phase Change Memories With Optimized Ge_x_Sb_y_Te_z_. IEEE Trans. Electron. Devices.

[12] Privitera S.M.S., López García I., Bongiorno C., Sousa V., Cyrille M.C., Navarro G., Sabbione C., Carria E., Rimini E. (2020). Crystallization Properties of Melt-Quenched Ge-Rich GeSbTe Thin Films for Phase Change Memory Applications. J. Appl. Phys..

[13] Redaelli A., Petroni E., Annunziata R. (2022). Material and Process Engineering Challenges in Ge-Rich GST for Embedded PCM. Mater. Sci. Semicond. Process..

[14] Tae Kim Y., Kim S.-I. (2013). Comparison of Thermal Stabilities between Ge-Sb-Te and In-Sb-Te Phase Change Materials. Appl. Phys. Lett..

[15] Morikawa T., Kurotsuchi K., Kinoshita M., Matsuzaki N., Matsui Y., Fujisaki Y., Hanzawa S., Kotabe M., Moriya H., Iwasaki T. Doped In-Ge-Te Phase Change Memory Featuring Stable Operation and Good Data Retention. Proceedings of the 2007 IEEE International Electron Devices Meeting.

[16] Fallica R., Stoycheva T., Wiemer C., Longo M. (2013). Structural and Electrical Analysis of In Sb Te-based PCM cells. Physica status solidi (RRL). Rapid Res. Lett..

[17] Selmo S., Cecchini R., Cecchi S., Wiemer C., Fanciulli M., Rotunno E., Lazzarini L., Rigato M., Pogany D., Lugstein A. (2016). Low Power Phase Change Memory Switching of Ultra-Thin In_3_Sb_1_Te_2_ Nanowires. Appl. Phys. Lett..

[18] Cecchini R., Selmo S., Wiemer C., Rotunno E., Lazzarini L., De Luca M., Zardo I., Longo M. (2018). Single-Step Au-Catalysed Synthesis and Microstructural Characterization of Core–Shell Ge/In–Te Nanowires by MOCVD. Mater. Res. Lett..

[19] Chèze C., Righi Riva F., Di Bella G., Placidi E., Prili S., Bertelli M., Diaz Fattorini A., Longo M., Calarco R., Bernasconi M. (2022). Interface Formation during the Growth of Phase Change Material Heterostructures Based on Ge-Rich Ge-Sb-Te Alloys. Nanomaterials.

[20] Nolot E., Sabbione C., Pessoa W., Prazakova L., Navarro G. (2021). Germanium, Antimony, Tellurium, Their Binary and Ternary Alloys and the Impact of Nitrogen: An X-Ray Photoelectron Study. Appl. Surf. Sci..

[21] Klein A., Dieker H., Späth B., Fons P., Kolobov A., Steimer C., Wuttig M. (2008). Changes in Electronic Structure and Chemical Bonding upon Crystallization of the Phase Change Material GeSb_2_Te_4_. Phys. Rev. Lett..

[22] Baeck J.H., Ann Y., Jeong K.-H., Cho M.-H., Ko D.-H., Oh J.-H., Jeong H. (2009). Electronic Structure of Te/Sb/Ge and Sb/Te/Ge Multi Layer Films Using Photoelectron Spectroscopy. J. Am. Chem. Soc..

[23] Kim J.-J., Kobayashi K., Ikenaga E., Kobata M., Ueda S., Matsunaga T., Kifune K., Kojima R., Yamada N. (2007). Electronic Structure of Amorphous and Crystalline (GeTe)_1−x_(Sb_2_Te_3_)_x_ Investigated Using Hard X-ray Photoemission Spectroscopy. Phys. Rev. B.

[24] Lee Y.M., Jung M.-C., Shin H.J., Kim K., Song S.A., Jeong H.S., Ko C., Han M. (2010). Temperature-Dependent High-Resolution X-Ray Photoelectron Spectroscopic Study on Ge_1_Sb_2_Te_4_. Thin Solid Films.

[25] Sosso G.C., Caravati S., Gatti C., Assoni S., Bernasconi M. (2009). Vibrational Properties of Hexagonal Ge_2_Sb _2_Te_5_ from First Principles. J. Phys. Condens. Matter.

[26] Abou El Kheir O., Dragoni D., Bernasconi M. (2021). Density Functional Simulations of Decomposition Pathways of Ge-Rich GeSbTe Alloys for Phase Change Memories. Phys. Rev. Mater..

[27] Abou El Kheir O., Bernasconi M. (2021). High-Throughput Calculations on the Decomposition Reactions of Off-Stoichiometry GeSbTe Alloys for Embedded Memories. Nanomaterials.

[28] Zuliani P., Palumbo E., Borghi M., Dalla Libera G., Annunziata R. (2015). Engineering of Chalcogenide Materials for Embedded Applications of Phase Change Memory. Solid-State Electron..

[29] Bragaglia V., Holldack K., Boschker J.E., Arciprete F., Zallo E., Flissikowski T., Calarco R. (2016). Far-Infrared and Raman Spectroscopy Investigation of Phonon Modes in Amorphous and Crystalline Epitaxial GeTe-Sb_2_Te_3_ Alloys. Sci. Rep..

[30] Kumar A., Cecchini R., Wiemer C., Mussi V., De Simone S., Calarco R., Scuderi M., Nicotra G., Longo M. (2021). Phase Change Ge-Rich Ge–Sb–Te/Sb_2_Te_3_ Core-Shell Nanowires by Metal Organic Chemical Vapor Deposition. Nanomaterials.

[31] Di Biagio F., Cecchi S., Arciprete F., Calarco R. (2019). Crystallization Study of Ge-Rich (GeTe)*_m_*(Sb_2_Te_3_) *_n_* Using Two-Step Annealing Process. Phys. Status Solidi RRL.

[32] Lannin J.S., Maley N., Kshirsagar S.T. (1985). Raman scattering and short range order in amorphous germanium. Solid State Commun..

[33] Kazimierski P., Tyczkowski J., Kozanecki M., Hatanaka Y., Aoki T. (2002). Transition from Amorphous Semiconductor to Amorphous Insulator in Hydrogenated Carbon−Germanium Films Investigated by Raman Spectroscopy. Chem. Mater..

[34] Jamali H., Mozafarinia R., Eshaghi A. (2017). The Effect of Carbon Content on the Phase Structure of Amorphous/Nanocrystalline Ge_1−x_C_x_ Films Prepared by PECVD. Surf. Coat. Technol..

[35] Prazakova L., Nolot E., Martinez E., Rouchon D., Fillot F., Bernier N., Elizalde R., Bernard M., Navarro G. (2022). The Effect of Ge Content on Structural Evolution of Ge-Rich GeSbTe Alloys at Increasing Temperature. Materialia.

[36] Cecchi S., Lopez Garcia I., Mio A.M., Zallo E., Abou El Kheir O., Calarco R., Bernasconi M., Nicotra G., Privitera S.M.S. (2022). Crystallization and Electrical Properties of Ge-Rich GeSbTe Alloys. Nanomaterials.

[37] Goriparti S., Miele E., Scarpellini A., Marras S., Prato M., Ansaldo A., DeAngelis F., Manna L., Zaccaria R.P., Capiglia C. (2014). Germanium Nanocrystals-MWCNTs Composites as Anode Materials for Lithium Ion Batteries. ECS Trans..

[38] Sousa V., Navarro G., Castellani N., Coue M., Cueto O., Sabbione C., Noe P., Perniola L., Blonkowski S., Zuliani P. Operation Fundamentals in 12Mb Phase Change Memory Based on Innovative Ge-Rich GST Materials Featuring High Reliability Performance. Proceedings of the 2015 Symposium on VLSI Technology (VLSI Technology).

[39] Kiouseloglou A., Navarro G., Sousa V., Persico A., Roule A., Cabrini A., Torelli G., Maitrejean S., Reimbold G., De Salvo B. (2014). A Novel Programming Technique to Boost Low-Resistance State Performance in Ge-Rich GST Phase Change Memory. IEEE Trans. Electron. Devices.

